# The Impact of Water, Sanitation and Hygiene Interventions to Control Cholera: A Systematic Review

**DOI:** 10.1371/journal.pone.0135676

**Published:** 2015-08-18

**Authors:** Dawn L. Taylor, Tanya M. Kahawita, Sandy Cairncross, Jeroen H. J. Ensink

**Affiliations:** 1 Environmental Health Group, London School of Hygiene and Tropical Medicine, Keppel Street, London, WC1E 7HT, United Kingdom; 2 Medecins Sans Frontieres/Artsen Zonder Grenzen, Plantage Middenlaan 14, 1001 EA, Amsterdam, The Netherlands; The Hospital for Sick Children, CANADA

## Abstract

**Background and Methods:**

Cholera remains a significant threat to global public health with an estimated 100,000 deaths per year. Water, sanitation and hygiene (WASH) interventions are frequently employed to control outbreaks though evidence regarding their effectiveness is often missing. This paper presents a systematic literature review investigating the function, use and impact of WASH interventions implemented to control cholera.

**Results:**

The review yielded eighteen studies and of the five studies reporting on health impact, four reported outcomes associated with water treatment at the point of use, and one with the provision of improved water and sanitation infrastructure. Furthermore, whilst the reporting of function and use of interventions has become more common in recent publications, the quality of studies remains low. The majority of papers (>60%) described water quality interventions, with those at the water source focussing on ineffective chlorination of wells, and the remaining being applied at the point of use. Interventions such as filtration, solar disinfection and distribution of chlorine products were implemented but their limitations regarding the need for adherence and correct use were not fully considered. Hand washing and hygiene interventions address several transmission routes but only 22% of the studies attempted to evaluate them and mainly focussed on improving knowledge and uptake of messages but not necessarily translating this into safer practices. The use and maintenance of safe water storage containers was only evaluated once, under-estimating the considerable potential for contamination between collection and use. This problem was confirmed in another study evaluating methods of container disinfection. One study investigated uptake of household disinfection kits which were accepted by the target population. A single study in an endemic setting compared a combination of interventions to improve water and sanitation infrastructure, and the resulting reductions in cholera incidence.

**Discussion and Recommendations:**

This review highlights a focus on particular routes of transmission, and the limited number of interventions tested during outbreaks. There is a distinct gap in knowledge of which interventions are most appropriate for a given context and as such a clear need for more robust impact studies evaluating a wider array of WASH interventions, in order to ensure effective cholera control and the best use of limited resources.

## Introduction

Cholera is a diarrhoeal disease caused by infection with the bacteria *Vibrio cholera*. It is a water- and foodborne disease with person-to-person transmission resulting from poor hygiene, limited access to sanitation, and inadequate water supply, which all contribute to the rapid progression of an outbreak.

Cholera outbreaks can occur during emergencies, such as earthquakes and flood events, or in refugee settings when water supply, sanitation and hygiene (WASH) infrastructure is compromised. The World Health Organisation (WHO) estimates that there are between 3–5 million cholera cases and 100,000–120,000 deaths every year, of which only a fraction are officially reported [1]. In 2013, 129,064 cases and 2,102 deaths were reported worldwide, with 44% of cases reported in Africa, and 45% in Haiti alone, where as of December 2013, 696,794 cases have been reported with 8,531 deaths since the outbreak began [2].

When an outbreak is detected the WHO recommends a response focussing on reducing mortality by ensuring prompt case management, and reducing morbidity by providing safe water, adequate sanitation and health promotion (for improved hygiene and safe food handling practices) for the affected community [1]. Consequently cholera epidemics require the same interventions used to prevent and control diarrhoeal diseases. The first responders to an outbreak will generally employ activities such as water trucking of chlorinated water, chlorinating individual water containers or distribution of products for household water treatment. This will most likely be accompanied by personal and food hygiene promotion as well as household disinfection and hygiene kit distribution. Due to the need for a quick response to contain the spread of the outbreak, multiple interventions may be implemented at the same time as community education, but this can be potentially limited according to the responder’s resources and capacity.

Despite a wide body of evidence investigating the effectiveness of WASH interventions against endemic diarrhoeal disease, many studies are considered to be of poor quality and the relative impact of each separate WASH intervention remains a contentious issue [3–5]. Assessing the impact of WASH interventions is challenging due to methodological issues; for example, a blinded trial of sanitation is impossible because people cannot be induced to use a toilet without their knowledge. The alternatives—blinding the subjects to the choice of outcome, has been used relatively rarely and blinding of outcome assessors or data analysts is not used enough. A further challenge relates to epidemiological issues where for example, improvements in the amount of water available will likely also have an impact on water quality and hygiene in the household.

A systematic review and meta-analysis evaluating the impact of WASH interventions on diarrhoeal disease concluded that each intervention type had a similar degree of effect and that water quality interventions were more effective than previously considered [6]. This was subsequently challenged when water quality studies were shown to suffer from bias and other methodological flaws [5, 7, 8].

The discussion about the relative importance of each intervention seems trivial, as ideally for diseases like cholera, with multiple transmission routes, all interventions should be implemented simultaneously. However during outbreaks in resource-poor settings, choices need to be made as to which intervention should, and can be implemented in order to have the greatest impact. To inform further research, policy and practice, a systematic review was conducted on the current evidence for uptake, use and health impact of WASH interventions to control cholera.

## Methods

The guidelines for the Preferred Reporting Items for Systematic Reviews and Meta-Analyses (PRISMA) were used to conduct a systematic search for original research of the impact of WASH interventions implemented to control cholera in a low, or middle income country [9]. The key inclusion criteria were: i) a clearly defined WASH intervention, ii) a cholera health outcome, or data pertaining to the function and use of the WASH intervention. Experimental, observational and qualitative studies were considered for inclusion when they clearly described a cholera outbreak or endemic setting and were published in peer-reviewed journals. Reports of previous systematic reviews, letters to editors and epidemiological investigations of outbreaks were excluded. Furthermore case-control studies which only examined risk factors for the disease, and not interventions to control the disease, were excluded as they would not add evidence to this particular review.

Four main concepts; water supply, water quality, sanitation and hygiene, were searched in combination with cholera and associated synonyms of acute watery diarrhoea, and with low or middle income countries as defined by the World Bank. The search strategy is presented in S1 Table. The search was conducted in November 2013 of five online databases: Medline, Global Health and Embase (through Ovid SP), Web of Knowledge and Africa Wide Information. In addition the bibliographies of recent evidence reviews were hand-searched for additional references [10, 11]. Furthermore any articles arising from web alerts using the same search strategy up to 31 December 2013 were considered. All records were entered in to EndNote X6 (Thomson Reuters, New York, USA) and duplicates removed. Titles and abstracts were then screened for eligibility followed by full review of the final selection of articles.

Studies were initially categorised according to whether measurement of a statistical association between the intervention and a health outcome had been made (Category A), whether only a change in a health outcome was reported (Category B) or where this was not measured at all (Category C). A methodological quality assessment of the included studies was then conducted based on the STROBE and CONSORT standards (S1 Checklist) for observational and trial studies respectively [12, 13]. This was supplemented using the review by Blum and Feachem, on measuring the impact of WASH investments on diarrhoeal diseases [14], which assessed methodological weaknesses; i.e. lack of adequate control groups, one to one comparison, inadequate control for confounding variables, health indicators recall, health indicator definition, failure to analyse by age and failure to record facility usage. Studies were then classified according to the WASH intervention implemented and relevant data extracted. Where more than one intervention was implemented, studies were classified according to the dominant intervention based on the amount of data presented.

## Results

**Fig 1:**
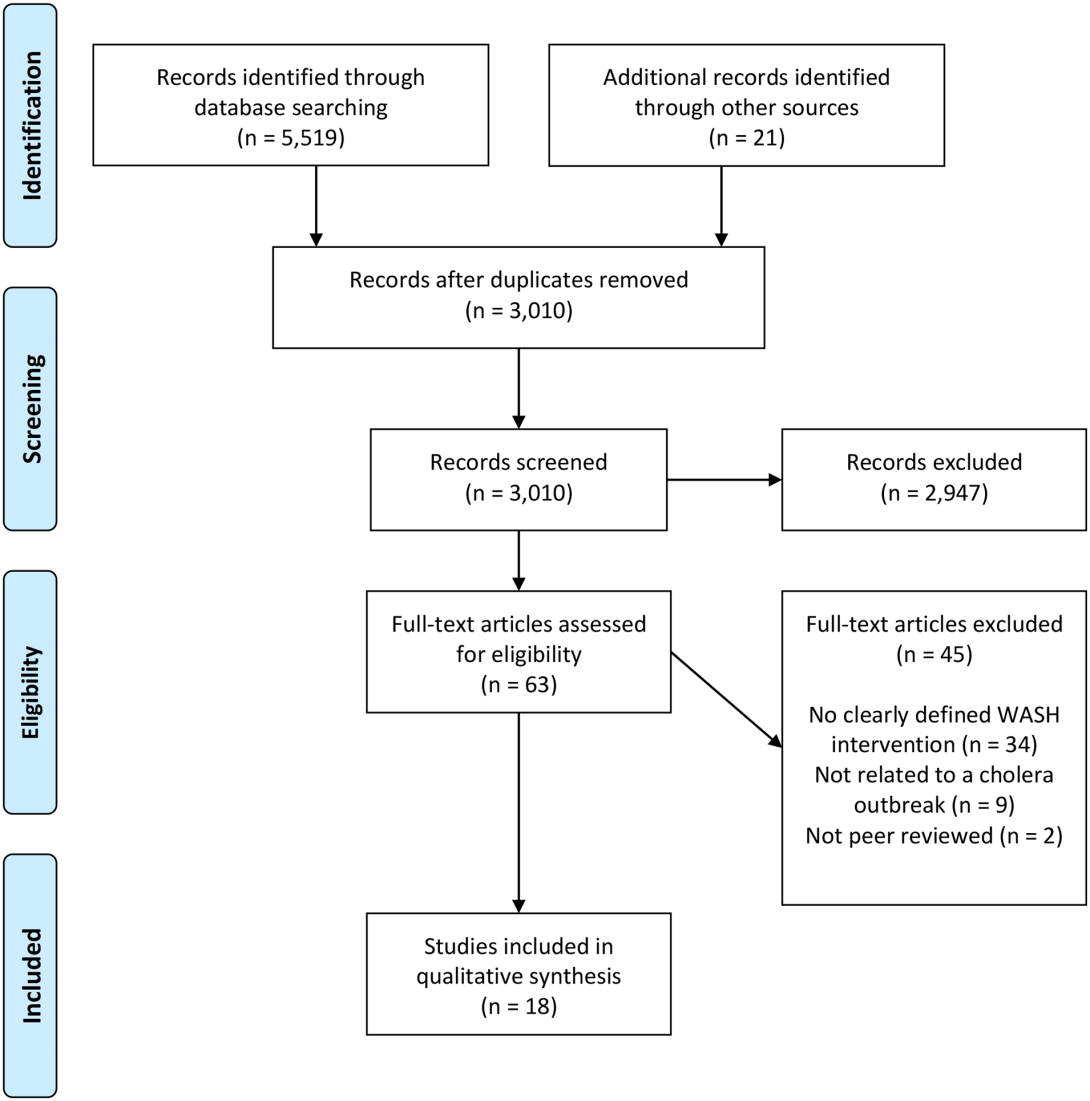
PRISMA flow diagram for literature search.

A total of 5,519 citations were identified from Medline (1,092), Global Health (1,801) Embase (1,467) Web of Knowledge (374) and Africa Wide Information (785), and a further 21 from hand-searches and recommendations of articles submitted for publication. After removal of duplicates (2,530), screening of 3,010 titles and abstracts resulted in the exclusion of 2,947 papers. Content review of 63 full texts led to the exclusion of an additional 45 papers. The main reasons for exclusion were; no clearly defined WASH intervention (34), not related to cholera (9), or not peer-reviewed (2). Finally a total of 18 papers were included in the systematic review (Fig 1).

Only 22% (4/18) of intervention studies conducted a test for statistical significance of any association between WASH interventions and a health outcome (Category A). One paper reported a change in health outcome without a statistical association (Category B). The remainder simply reported usage, or outputs of interventions (Category C). Using further quality assessment, no paper was deemed high quality, 44% were considered medium quality leaving the majority of low quality papers (Table 1). The quality assessment criteria used are presented in S2 Table.

**Table 1:** Data extraction of studies included in the review.

Study	Intervention	Country, Setting	Study design	Outcome Measure	Sample Size	Results	Category, Quality
**Water treatment at source**							
Cavallaro et al. (2011)	Pot chlorination of wells. Pierced plastic bottle with layers of sand, gravel and calcium hypochlorite.	Guinea-Bissau, cholera outbreak	Intervention trial	FRC	30 wells in 22 Bissau neighbourhoods affected by cholera	WHO recommended emergency FRC level for outbreaks (>1mg/l) maintained in 15% of wells at 24 hours. No measurement of pH or turbidity, abstraction rate, rainfall or indicator bacteria. Pot chlorination is ineffective and should be discouraged.	C, LOW
Garandeau et al. (2006)	Well chlorination devices. Floating chlorinator, locally made pot chlorinator, pressed hypochlorite in sand filled pierced plastic bag.	Liberia, cholera outbreak	Intervention trial	FRC	12 public wells in 3 peri-urban communities of Monrovia hosting displaced people	No numerical results presented. Statements on effectiveness (FRC remaining between 0.2–1.0mg/l) and appropriateness (availability of local materials and acceptability) for each device trialled. Locally pressed calcium hypochlorite tablets in pierced plastic bags together with adequate training can be effective.	C, LOW
Guevart et al. (2008)	Well chlorination device. Hypochlorite and river sand in pierced plastic bag.	Cameroon, cholera outbreak	Intervention trial	FRC	18 wells in 2 Douala neighbourhoods	Maximum chlorine level reached after 24 hours in 31 out of 35 wells. On Day 4 the FRC was <0.2mg/l in half the wells. Presentation of results only, no analysis.	C, LOW
**Water treatment at point of use**							
Colwell et al. (2003)	Filtration. Intervention: Sari and nylon filters. Control: no filter.	Bangladesh, endemic cholera	Non-random controlled trial	Cholera incidence	133,000 people in rural Matlab randomised to sari (27 villages), nylon (25 villages) or control (13 villages).	Sari compared to control—48% reduction cholera. (p<0.001). Nylon compared to control—41% reduction cholera (p<0.02). No significant difference between nylon and sari groups.	A, MED
Conroy et al. (2001)	Solar disinfection. Intervention: 1.5L plastic bottles kept on roof. Control: water kept indoors.	Kenya, cholera outbreak	Randomised controlled trial, retrospective	Cholera incidence	155 and 144 Maasai children <6yrs randomised to solar disinfection (67 household) or control (64 households)	Cholera in invention group compared to control group: Adults: RR 1.2, 95% CI 0.59–2.5. Children aged 6–15: RR 1.09, 95% CI 0.58–2.05. Children <5 years: OR 0.12 (0.02–0.65) p = 0.014.	A, MED
Deb et al. (1986)	Household water treatment, safe storage. Interventions: Chlorine tablets; narrow mouthed storage container (‘sorai’); Control: nothing.	India, endemic cholera	Non-random controlled trial	Cholera incidence	91 families of index cases, (31, 30, 30) residing in Calcutta slums.	Cholera incidence reduced by 75% (p<0.001) in the storage container group compared to control. Cholera incidence reduced by 58% (p<0.01) in the chlorination group compared control group. Mean FRC in chlorination group was 0.2mg/l.	A, MED
Dunston et al. (2001)	Safe Water System. 0.5% sodium hypochlorite, narrow mouthed jerry can and education.	Madagascar, cholera outbreak and cyclones	Intervention trial	FRC, Chlorine product utilisation rate	375 households in 15 Antananarivo neighbourhoods	Utilisation rate of chlorine 11% after 6 months. Stratified by the stage of the mobilisation process completed. Median FRC in households using products and those not, 0.23mg/l, 0.1mg/l respectively (p = 0.005).	C, LOW
Huq et al. (2010)	Filtration. Sari filtration.	Bangladesh, endemic cholera	Cross-sectional study	Use of filter, Cholera incidence	7,470 rural women in Matlab	Five year after original trial, 31% reported using a filter of any type with 60% of those using a folded sari filter. 25% reduction in cholera incidence, not statistically significant.	A, MED
Lantagne & Clasen (2012)	Household water treatment, safe storage (HWTS). Kenya: chlorine tablets, flocculant/disinfectant—NFI distribution, Nepal: liquid chlorine and tablets—continuous distribution	Kenya, flood & cholera. Nepal, cholera outbreak	Cross-sectional study	Effective use, Coliform count,(CFU/10ml), FRC	400 households in Nepal, 409 households in Kenya	Confirmed use of HWTS method: Kenya– 11.7%, Nepal– 18.5%. Effective use at 1 CFU/100ml breakpoint: Kenya—Aquatabs, PuR– 5.3%, 2.3%. Nepal—not measured, unable to incubate at 44°C. Data compared with other emergencies—Targeted intervention more effective than NFI distribution where population is familiar with method.	C, LOW
Patrick, Berendes et al. (2013)	Household water treatment. Mass distribution of chlorine based products.	Haiti, cholera outbreak	Cross-sectional study	FRC & bacterial colony counts	433 households in 37 clusters in Artibonite, 108 water samples	51% improved sources contaminated with E.coli. 81% respondents reported treating water in the past 3 months. 32% households had a water treatment product present. 49% of respondents reported using an acceptable dose of Aquatabs. 16% of respondents reported using an acceptable dose of liquid bleach. 13% of respondents with water to test sample had a detectable FRC.	C, MED
Quick, Venczel et al. (1996)	Chlorine solution, storage vessel. Group A: Vessel + chlorine, Group B: Vessel, Group C: Control	Bolivia, cholera outbreak	Non-random controlled trial	FRC & bacterial colony counts	42 (A-15, B-15, C-12) households in El Alto collecting water from shallow wells.	Baseline: 93% of wells and 79% usual vessels had faecal coliforms present. Intervention: Geometric mean faecal coliform and E coli count substantially lower in group A compared to B and C. Households in group A had mean FRC at 1^st^ 2^nd^ and 3^rd^ visits of 1.6, 1.2 and 0.7mg/l respectively.Follow-up: 73% samples had detectable chlorine, 60% no faecal coliforms detected, 78% no E.coli colonies detected.	C, MED
**Hygiene promotion**							
Beau De Rochars et al. (2011)	Cholera prevention campaign	Haiti, cholera outbreak	Cross-sectional study	Cholera awareness, FRC, Coliform count,	405 household heads in 27 clusters in resource limited areas, in and near Port au Prince.	Preferred method of messaging: TV 71%, Radio 69%. Knowledge of cholera symptoms: Diarrhoea (89%), vomiting (83%). Knowledge of transmission: Contaminated water (72%), contaminated food (61%). Cholera awareness: 86% mentioned hand washing as prevention method. 94% reported washing their hands with soap. Water treatment practices increased from 30% before to 74% after the outbreak (p<0.05). 66.6% used water purification tablets, 57.7% used bleach. 64% water sources positive for E.coli with 60% stored water samples showed detectable FRC.	C, MED
Einarsdottir et al. (2001)	Health education	Guinea-Bissau, cholera outbreak	Cross-sectional study	Cholera awareness	53 residents in 1 village in Biombo region	No one could explain the transmission of cholera. 94% reported hearing at least one prevention message. 68% of those who reported, could state one prevention method. 45% heard cholera information on the radio of which 63% could explain at least one prevention method. 41% heard information by word of mouth of which 64% could explain at least one prevention method. 66% consumed water with lemon in it. 40% consumed boiled water. Radio and word of mouth important dissemination methods. High acceptance for using lemon to treat water.	C, LOW
Mahadik & Mbomena (1983)	Health education	Zambia, cholera outbreak	Cross-sectional study	Cholera awareness	573 (442 intervention, 131 control) household heads in 16 intervention and 2 control villages in Luapula province	99% and 92% of respondents demonstrated cholera awareness in the intervention and control arms respectively. Unclear how this result was obtained from the data. Results where comparison group had a higher level of awareness than intervention group are not addressed. Inconclusive results.	C, LOW
Quick, Gerber et al. (1996)	National cholera prevention campaign	Peru, cholera outbreak	Cross-sectional study	Cholera awareness	132 (67 urban, 65 rural), household heads in Amazon communities	93% of rural and 67% of urban respondents believed they could prevent cholera transmission. Sources of cholera information: Urban areas: radio (71%), TV (64%) and group talk (50%). Rural areas: health workers (70%), gov’t authority (61%) and radio (58%).	C, LOW
**Water storage vessel disinfection**							
Steele et al. (2008)	Disinfection of jerry can with sodium hypochlorite	Uganda, cholera outbreak	Cross-sectional study	Coliform count	13 households in Kitgum displaced camp	Water sources not contaminated. Methods used were effective for cleaning of jerry cans but did not prevent recontamination. Raw data presented but no analysis.	C, LOW
**Household disinfection**							
Gartley et al. (2013)	Household disinfection kit distribution	Haiti, cholera outbreak	Cross-sectional study	Uptake and use of kits	208 recipient households in Carrefour	98% of households had used the kit. 75% used 5 or more items. Most popular was chlorine and soap. Increased use observed after hygiene education messages strengthened.	C, LOW
**Improved WASH infrastructure**							
Azurin & Alvero (1974)	Improved water and sanitation facilities. Intervention: Improved water, improved communal toilets, improved water & toilets, Control: none	Philippines, endemic cholera	Non-random controlled trial	Cholera incidence	4 communities in Bacolod city	Compared to the control community: 68% reduction in cholera incidence rate in community with improved sanitation. 73% reduction in cholera incidence rate in community with improved water supply. 65% reduction in cholera incidence rate in community with improved water and sanitation. Cholera is less likely to produce secondary cases where improved WASH measures are in place.	B, MED

The eighteen papers identified, presented research from Africa (48%), Latin America/Caribbean (26%) and Asia (26%), and included one paper presenting results from both Africa and Asia. Eleven studies (61%) investigated water quality interventions; those at source (17%) and those at point of use (44%). Four studies (22%) examined hygiene promotion, and three separate studies evaluated storage vessel disinfection, household disinfection and improvements to water and sanitation infrastructure (Table 1).

### Water treatment at source

Three intervention trial studies in Africa conducted preliminary investigations into well chlorination [15–17]; and were all assessed as low quality, primarily because they only presented descriptive water quality data without any statistical analysis. Well chlorination using liquid bleach was assessed following its implementation during the 2008 cholera outbreak in Guinea-Bissau [15]. The intervention was considered ineffective in maintaining the WHO recommended emergency level (>1mg/l) of free residual chlorine (FRC), with only 15% of inspected wells having met the standard 24 hours following chlorination. The study further reported that families had discontinued their household water treatment following well chlorination, as they believed their water to be safe. This action may have paradoxically increased their risk of cholera, and the paper concluded that the intervention was an ineffective, costly and impractical method of water treatment and should be discouraged.

The two remaining studies evaluated chlorinators; chlorine-containing devices that when suspended in well water gradually release chlorine leaving a continuous FRC [16, 17]. Both studies demonstrated the functionality of locally constructed chlorinators as adequate FRC could be maintained for approximately 3 days, though the studies did not report what the maximum period was, under which the devices could guarantee this. Both studies were preliminary investigations, and health education and training related to the use of chlorinators was not provided. Only one of the studies [16] evaluated the appropriateness of the chlorinators according to the local availability of materials, the ease of operation and maintenance, the acceptability by communities, the cost and logistic issues. The study found an effective and appropriate chlorinator by using a pierced plastic bag filled with sand with a locally pressed chlorine tablet placed at its centre. Well users apparently found the system both easy to use and maintain, however as no follow-up data was presented it is difficult to draw conclusions on its long term effectiveness and acceptability.

### Household water treatment (HWT)

Eight studies presented a point of use water quality intervention; which included: filtration (2), solar disinfection (1) and chlorination products with, or without storage vessels (5).

#### Filtration

Two linked studies from Bangladesh evaluated, and followed-up an intervention using sari cloth to filter surface water [18, 19]. A non-randomised controlled trial was conducted from 1999 to 2002, where clusters of villages were allocated to receive saris, or nylon cloths to filter surface water, and compared to control clusters. Cholera incidence data was obtained through hospital records. Results of the trial suggested that cholera incidence was reduced by 48% in the sari filter group (p<0.001), and 41% in the nylon filter group (p<0.02) when compared to the control group. However, the water sources used by households varied, and it was unclear if households had used tube well, or surface water. Additionally, the type of water storage in the home was not investigated. In the follow-up survey 5 years later, 31% of respondents interviewed reported still using a filter, with 60% of those using a sari filter. The study reported a 25% reduction in cholera incidence over the evaluation period, though the difference was not statistically significant.

#### Solar disinfection

A study conducted among the Maasai in Kenya evaluated the impact of solar disinfection (SODIS) using the number of self-reported cholera cases over a three month period following an outbreak [20]. This study was a follow-up to a previous study in which households with children under the age of five had been randomised to SODIS [21]. All previously selected households were visited within six weeks of the outbreak, and local criteria for case definition were used to identify cholera cases. No significant difference in cholera incidence between intervention and control groups was found for those aged over five years. However, the odds of cholera in those <5 years were 88% lower in the SODIS group when compared to the control group (p = 0.014). However, actual use of and compliance with the intervention by the population was not assessed.

#### Chlorination with or without safe storage vessels

The most published WASH intervention was chlorination of water stored in the household, investigated by five studies. The intervention consisted either of the distribution of chlorine products alone, or in combination with safe water storage vessels.

A trial carried out in an endemic setting in India, randomised family contacts of 91 hospital admitted cholera patients to a study arm that received either: chlorine tablets (1.25mg/litre of water), a local narrow-necked storage container, or acted as a control arm [22]. Presence of *Vibrio cholerae* was confirmed in bacteriological tests in participants for up to 5 days after identification of the index case. Results of this testing showed that the incidence of cholera infection was reduced by 75% (p<0.001) and 58% (p<0.01) in the storage container and chlorination groups respectively when compared to the control group.

The Safe Water System developed by the US Centers for Disease Control and Prevention (CDC), comprises behaviour change techniques, alongside point of use treatment and safe storage of water. Two studies evaluated the safe water system. A programme in Antananarivo, Madagascar implemented in response to a major cholera outbreak, used community mobilisation and social marketing of a locally produced 0.5% solution of sodium hypochlorite (Sûr’Eau) and distributed product use information as well as a 20 litre jerry can [23]. Baseline and follow-up surveys found an average utilisation rate of 11.2% after 6 months, which ranged from to 8.4% to 19.7% depending on whether communities were in an early, or final stage of the mobilisation process. The FRC level in stored water was 0.23mg/l in households using the product compared to 0.10mg/l in those not using it (p = 0.005).

A trial in El Alto, Bolivia systematically selected 42 households of 55 community volunteers, who were then randomised to receive either; i) 0.5% calcium hypochlorite solution and a 20 litre narrow-mouthed jerry can with information on use, ii) a jerry can only or iii) nothing [24]. During post intervention sampling, over 85% of water samples from households receiving the full intervention had detectable levels of FRC, reaching 100% at final sampling. Pre-intervention, 93% of wells and 79% of water containers were found to be positive for faecal coliforms. Post-intervention contamination was found to be substantially lower in the samples from households receiving the full intervention compared to the others. A further evaluation three months post-intervention observed that over 73% of samples had detectable FRC levels, and were free from faecal contamination.

One study investigated distribution of chlorine products only. Results from cholera outbreaks in Kenya and Nepal were extracted from a four-country study [25]. Outcomes were: reported use of the product, confirmed use (detectable FRC) and effective use (water samples <1 CFU or *E*. *coli*/100 ml). In Nepal, chlorine tablets (Aquatabs) and liquid chlorine (Piyush, Waterguard) were continuously distributed to 1,565 homes in 2 sub-districts. Confirmed use was limited to 18.5% of households and effective use was not measured. In Kenya, chlorine tablets (Aquatabs) and flocculant/disinfectant (PuR) were distributed as part of kits to 5,592 homes in 4 communities. Confirmed use was 11.7% and effective use of Aquatabs and PuR was just 5.3% and 2.3% respectively.

The most recent study evaluated the response to the Haiti cholera epidemic in 2010, where the government and other agencies implemented a free mass distribution of various chlorine products alongside hygiene promotion activities in a rural setting [26]. The majority of households (81%) reported having treated water in the past few months, however only 32% had any treatment product present in the house, primarily due to problems of affordability and access. The most popular treatment product used by those reporting was Aquatabs (86%), followed by liquid bleach (24%). 49% and 16% of respondents reported using an acceptable dose of Aquatabs or liquid bleach respectively, with the remainder either under dosing or not knowing the correct dosage. This translated into just 13% of samples having a detectable FRC.

### Hygiene promotion

Four cross-sectional studies, two in Africa and two in Latin America, were identified as evaluating community knowledge, and awareness of cholera prevention, and all reported a positive effect.

One assessed the effectiveness of interventions implemented during the 2010 cholera outbreak in Haiti, which included mass media campaigns, community health worker activities, and distribution of water purification tablets, soap and oral rehydration solution [27]. The preferred method of communication of those receiving cholera messages was TV (71%) and radio (69%). Knowledge of common symptoms was high (>80%) and 86% of respondents cited hand washing as a prevention method. Water treatment practices increased from 30% before the outbreak to 74% after the outbreak (p<0.05). The most common methods were water purification tablets (67%) and bleach (58%). The majority (64%) of water sources tested positive for *E*. *coli*, but 60% of stored water samples showed detectable FRC levels. The results of the survey suggest that the public health messages were effective, and promoted behaviour changes with regard to water treatment practice.

A study in rural Guinea Bissau during the 1994 cholera outbreak explored local views about cholera and the diffusion of health messages, and assessed whether the messages contributed to behaviour change [28]. Interventions included messages to boil water, or drink water with lemon in it, wash hands with soap, take precautions with food, sweep the family compound, keep children away from dirt, keep flies away, and build and use latrines. The study reported that 94% of 53 interviewees had heard, or seen a cholera prevention message, and that 68% of those, recalled at least one preventative method. Radio was the most common source of information (45%) followed by word of mouth at 41%. While 70% of the respondents would consume boiled water, or water with lemon juice added to it, not a single person could explain the transmission of cholera. The absence of baseline data made it impossible to draw a conclusion regarding a change in behaviour.

Mahadik reported that 99% of 442 respondents in 16 villages exposed to a cholera education programme in Zambia showed awareness of cholera compared to 92% of 131 respondents in two control villages in a different district, however no test of significance was reported [29]. The paper provided no information on how the results were reached, nor was there a description of the study population. Further results show that the control group had better knowledge of the main cholera transmission routes than the intervention group, but these were not discussed.

Quick et al. conducted a knowledge, attitudes and practice survey in urban and rural Amazon communities, to assess the impact of a nationwide prevention campaign, 6 months after the beginning of the Peruvian cholera epidemic in 1991 [30]. The study found that 93% of rural, and 67% of urban respondents believed they could prevent cholera transmission. Sources of cholera information in urban areas were radio (71%), TV (64%) and group health talks (50%) compared to rural areas where health workers (70%), government authorities (61%) and radio (58%) were the most common. Whilst knowledge and perceived importance of cholera prevention methods was high, this did not translate into practice. For example 88% of the urban group and 92% of the rural group knew that drinking treated water was a cholera prevention method but only 25% and 23% respectively, always practised this. Several limitations of the study were reported, including the groups not being representative of the population, use of different sampling methods and time constraints preventing the possibility of verifying reported responses through direct observation.

### Storage vessel disinfection

A study in a camp in Kitgum, Uganda (2007), assessed the effectiveness of jerry can cleaning using a strong disinfectant, and the recontamination potential after cleaning [31]. Two different methods were used to clean selected jerry cans from 13 households. The container was either half-filled with chlorine solution and shaken for 1 minute (9 jerry cans), or completely filled and allowed to sit for 1 minute (2 jerry cans) or 5 minutes (2 jerry cans), before all being rinsed and filled with source water (free of coliforms). Samples were taken for microbiological testing before cleaning, after cleaning and at 3 and 5 days thereafter. The coliform count was reduced in 85% of the cleaned jerry cans but no method was deemed more effective than the other. However more strikingly, these methods did not prevent recontamination, at the household, in 46% of the jerry cans.

### Household disinfection

One recent study measured the uptake of household disinfection kits as an additional prevention method in the Haiti cholera outbreak [32]. Disinfection kits comprising a 14 litre bucket, scrubbing brush, cloth, chlorine bleach, 10 litre jerry can and soap were distributed to the caretakers of patients admitted to a treatment centre, who also attended a hygiene promotion session. In a follow-up survey 98% of 208 households had already used the kit with the two most popular items being the chlorine and soap. A significant increase in use of the kits was observed after the date when hygiene promotion messages were strengthened. Households used the kits to clean floors (73%) and dishes (62%) followed by laundry and latrines. The study acknowledges several limitations; the lack of a control group, the sampling method may have influenced representativeness, and the lack of measurement of time of ownership of the kits may have biased the results.

### Improvements to WASH infrastructure

A study conducted in the Philippines attempted to evaluate the impact of improvements to WASH infrastructure on cholera incidence by comparing a control community with communities with: i) safe water supply, ii) shared toilet facilities, and iii) both water supply and shared toilet facilities [33]. Bacteriological surveillance of confirmed cholera, conducted from 1968–1972 suggested that cholera incidence could be reduced by 68%, 73%, and 76%, by implementing low cost sanitation, water supply or both, respectively. Unfortunately the study suffered from several key methodological shortcomings: i) one to one comparison of interventions implemented at the community level without controlling for intra-cluster correlation, effectively the same as comparing one person with just one other, ii) actual use and functionality of the interventions by the population was not assessed, nor were water quality or water use tests conducted, and iii) no statistical test was provided, nor was the data controlled for other risk factors.

## Discussion

The primary objective of this review was to identify and assess the evidence for the effectiveness of WASH interventions to control cholera, and provide recommendations to implementers during cholera outbreaks, while a secondary objective was to highlight the gaps in knowledge and identify areas for further research. This review presented findings from eighteen studies of which only five reported a health outcome. The studies were heterogeneous in their design, measurement of outcomes, contexts and interventions deeming a meta-analysis inappropriate. The review thus found a lack of good quality studies, while those studies that were included focussed predominantly on water quality interventions.

### Lack of evidence

The lack of good quality studies, with well defined health outcomes, and consequently a relaxing of exclusion criteria has left our search open to a number of potential biases and limitations which will have had an effect on the results reported. Whilst no time limit was set on the search, and hand searches were done of recent reviews for additional references a decision was made not to include unpublished literature. There is likely to be a wealth of useful information available in operational and evaluation reports by non-governmental organisations (NGOs) working in emergencies and cholera outbreaks, however it is unclear how much is available as not all is in the public domain, while some might contain sensitive information that NGOs are unwilling to share. The grey literature warrants a separate review, as it could provide valuable lessons learnt, not only regarding the approaches used, the types of intervention chosen and implemented, and their successes or failures, but could also provide valuable information regarding the setting in which the outbreaks occurred. Ideally a review should separate lessons learnt from natural disasters, and conflict responses, and distinguish between urban and rural settings, and of course between interventions implemented for those internally, or externally displaced, and those still living in their own housing. Currently the available literature does not allow for this distinction.

This study found a clear lack of evidence to help guide implementers decide what approach and intervention to select during a cholera outbreak. This is, to a very large extent, a result of the difficulties associated with collecting epidemiological evidence and time constraints during epidemics, which often occur during, and following natural disasters or conflicts. The main desire of those involved in cholera control is to limit the speed of transmission and to reduce mortality, whilst establishing baselines, and research in general, is often seen as taking valuable time and resources away from life-saving activities. There is further a belief held by some involved in emergency response that only randomized controlled trials (RCT) can provide evidence, and therefore that any study looking to gather evidence is going to be prohibitively expensive, time consuming and will require a lot of man power. Although RCTs are unlikely to be feasible in emergency settings, and could raise serious ethical concerns, this does not mean that good evidence cannot be collected. Most of the studies presented in this survey failed to adhere to the minimum evaluation procedures (MEP] recommended by the WHO for the evaluation of WASH studies [34], which recommends to measure functionality, adherence to interventions, and the use of intermediate outcomes like: water use, water quality and behavioural indicators, when disease data cannot be collected.

### Water source and household water quality interventions

The large majority of studies identified focussed on water quality interventions, even though no study provided evidence that the water quality route was the dominant route of transmission in the reported study areas. This seems to highlight the generally held belief that cholera is exclusively waterborne, thereby ignoring other routes of transmission. Since the cholera outbreaks in London in the 1850s, and the ground breaking work of John Snow, cholera seems to be synonymous with water quality, and the associated interventions. However, John Snow’s conviction that hand washing with soap could dramatically slow down, or even stop the rapid outbreak of cholera epidemics [35] is often forgotten.

Lakes, estuaries and to a lesser extent rivers are considered the main reservoir for V. *cholerae* and have been implicated in past outbreaks of cholera [36–38]. *V*. *cholerae* tends to favour slightly brackish to saline water, temperatures of up to 30°C and association with algae, zooplankton and copepods tends to promote its survival and pathogenicity [39]. These ‘water quality’ conditions tend not to prevail in well water, and wells are not considered reservoirs for cholera. Notwithstanding this, the studies of source water quality interventions in this review focussed exclusively on chlorination of public wells, possibly due to fear of local contamination by latrines or poor hygiene by those collecting water. However, as past studies have shown; water quality improvements at source are often ineffective as good quality source water becomes re-contaminated during collection or use within the household as a result of poor (hand) hygiene [40–42].

The well chlorination studies all reported problems with maintaining adequate levels of chlorine for prolonged periods, and finding an appropriate design that could be locally made and affordable. The acceptability of the devices and chlorinated water by the local population was not rigorously tested. Furthermore, interventions in Guinea-Bissau appeared to show that well chlorination without proper promotion and education led to a false sense of security [15, 43]. This highlights that water quality interventions are likely to be futile if not accompanied by adequate training, health education and hygiene promotion. In the case of well chlorination, implementers must be trained how to treat wells and monitor residual chlorine levels, while users must be informed of the contact time necessary for disinfection. Lastly and most importantly, hygienic water handling practices must be promoted as poor hygiene is likely to undermine an intervention as demonstrated in the study investigating methods of jerry can disinfection [31]. The findings suggest that well chlorination may be difficult to execute in emergency settings because of a lack of trained individuals, the number of wells within a given setting may be large, and unknown resulting in poor coverage, and the amount of chlorine required for each well may differ due to contamination with organic matter. In fact, the authors of the study in Guinea-Bissau clearly judged well chlorination to be an unsuitable intervention [15]. In light of this evidence we would not recommend well chlorination as a measure to control cholera.

Options for water treatment at the household level (HWT) were the most reported interventions in this review with distribution of chlorination products the most popular. However, half of the six studies that distributed a water quality intervention and conducted a health education programme, reported inconsistency of product use throughout the year, with chlorination in particular seen as an emergency measure, and used sporadically depending on its affordability and accessibility. Furthermore, mass distributed chlorine products were poorly used even where prevention knowledge was high [23, 25, 26]. Use and adherence of HWT is considered a challenge even during non-emergency settings [7]. Effective implementation of such interventions in emergencies remains a challenge, however it is clear that simply handing out chlorine, or other forms of HWT, and expecting sustainable behaviour change is unrealistic. For chlorination in particular, evidence from non-cholera emergencies indicates that effective use was highest where households with contaminated water were targeted, the treatment method effectively treated the water and the population was familiar with the method and was willing to use it [25]. Water quality interventions must be preceded by formative research and accompanied by health education so that appropriate products for HWT can be selected and sustainable behaviour change can be achieved. These lessons should be applied to future cholera interventions with more rigorous evaluation.

### Water treatment versus other WASH interventions

With cholera largely perceived as a waterborne infection, other transmission routes like the consumption of contaminated food as a result of poor hand hygiene, and person-to-person transmission appear to be overlooked in the literature. However, outbreaks in which waterborne transmission has not been implicated are plentiful. An extensive review of epidemiological data of cholera outbreaks in India, Bangladesh and the Philippines in the 1980s, found that 35–80% of cholera infection in the contacts of index cases occurred after more than 2 days, thereby suggesting that these contacts were infected by person-to-person transmission within the family [35]. The author further argues that even though introduction of cholera within a community is likely to be waterborne, transmission within the community is likely to occur through several routes at the same time. Hence HWT systems, as they focus exclusively on transmission via drinking water, are not by themselves, suitable interventions in every cholera outbreak and should not be employed as a universal remedy.

A review of WASH interventions in 1991 concluded that those for sanitation and water supply for improved hygiene yielded greater reductions in diarrhoeal disease than those for water quality [44]. This agreed with the conventional wisdom, and since then, sanitation and water supply interventions have largely dominated the focus of diarrheal disease prevention. However, since the beginning of this century there has been growing evidence in support of HWT [45]. Meta-analysis of HWT studies shows a combined reduction in diarrhoeal disease of 35% making it as effective on its own as improving access to water and sanitation [6]. These findings, as a result of study limitations, are certainly not without discordance [4, 5], and a more recent meta-analysis controlling for bias estimated that HWT could reduce diarrhoeal disease by a more modest 15% [8]. A recent review investigated the effect of hand washing with soap, water quality improvement and excreta disposal on reducing diarrhoeal disease [3] found that for HWWS, reductions of 42–48% were consistently shown, compared to interventions for water quality (17%) and excreta disposal (36%).

### Hygiene promotion and hand washing with soap

In light of the abundance of evidence supporting the promotion of hand washing to reduce diarrhoeal disease, and since the same WASH interventions apply to cholera it is surprising that so few studies specifically evaluate HWWS to prevent cholera. Only one study [27] presented results for self-reported use, and access to soap, which in itself can be subject to bias. The remaining studies [28–30] focussed on the effectiveness of the intervention in eliciting behaviour change and water treatment practices. It should also be appreciated from these findings that improved knowledge does not necessarily translate into improved practices, and as such there is a need to find ways to evaluate hand washing interventions in more depth. Even where hand washing has been ascertained by observation, the use of self-reported diarrhoea incidence as an outcome measure still introduces bias [5, 42]. In future, more objective outcomes such as pathogen presence will need to be used.

The reductions that meta-analysis have shown can be achieved, speak in support of HWWS as a cholera prevention measure, but more research and evaluation are needed to confirm its effectiveness. Furthermore, epidemiological investigations have reported a protective effect of hand washing when food was associated in cholera outbreaks, as well as in outbreaks where the source remained unidentified [46–48]. In an camp in Kenya during an outbreak investigation the source could not be identified, but the authors commented that the epidemic curve was not reflective of a point source outbreak, and found hand washing with soap to be protective [48]. In Zambia, an outbreak investigation identified the consumption of raw vegetables as the source of the epidemic, more precisely the poor hygiene practices of market vendors. Hand washing with soap was a strong protective factor, in contrast with disinfection of household water with a sodium hypochlorite solution [46]. We question the emphasis on water quality, when proper hand hygiene practice can prevent person-to-person transmission as well as food and household water contamination. Hygiene promotion, more precisely hand washing with soap, should be an integral component of any cholera control program. The promotion of HWWS requires formative research among the target population, and the development of tools to achieve and measure behaviour change, creating the illusion that it is expensive and time consuming. Given the rapid onset and high mortality associated with cholera outbreaks, water quality interventions are perceived as being the easiest, quickest and cheapest to deploy, especially when compared to sanitation and water supply programmes which require large investments in infrastructure. However, the distribution of soap is a simple and relatively affordable intervention that can be practised in the household while other interventions in the public domain are being executed [46], while good tools are available to help conduct formative research and plan behaviour change interventions for HWWS [49].

Evidence for diarrhoeal disease suggests that hygiene behaviour is sustained following implementation and is best delivered using small groups and frequent personal contact with a hygiene promoter [50]. The studies in this review all suggest that radio and TV are popular dissemination methods. Therefore the role of mass media should be explored further in comparison with more traditional methods, in those contexts where it is feasible.

### Intra-familial transmission

The study of disinfection of jerry cans [31] used for water transport and storage raises the question of the extent to which household hygiene practices must also be improved in order to reduce the excess risk of disease associated with contaminated water at the household level. Recontamination of water storage vessels will most likely be caused by members of the household bringing pathogens into the household and passing them to other family members.

In the past, it was a common practice in cholera outbreaks to spray the houses of cholera patients with disinfectant. This had the potential of causing damage to domestic property and stigmatising the patients and their families, putting in jeopardy the reporting and detection of cases and thus the whole outbreak control effort. Presently, household disinfection kits that normally include a bucket, bleach, soap, cloth and scrubbing brush, are increasingly being used as an alternative to household spraying. The distribution of kits places the responsibility with the family which reduces the stigma attached to the disease and may encourage sustained and improved hygiene at the household level [32]. There is limited evidence for the role of family contacts in the transmission of infection, although studies have found that having a case at home is a risk factor [51, 52] and that family members can spread *V*. *cholerae* in stored water and food through contaminated fingers [53]. The practice of sending spraying teams to disinfect houses of cholera patients is now discouraged as there is no evidence that one-off disinfection has any impact on transmission. Furthermore the resources required as well as the time delay before the disinfection is carried out make it less attractive as a practical intervention [54]. The use of kits should be evaluated further in order to inform future best practice.

## Conclusion

Several recent reviews [10, 55, 56] have highlighted a shortage of evidence for WASH interventions in emergencies, and all make recommendations for further research. This review found a lack of studies evaluating WASH interventions implemented to control cholera. The majority of studies lacked a disease outcome, or failed to assess compliance, or use of the intervention. There is without doubt a great need for studies evaluating cholera response interventions, in the spirit of the WHO Minimum Evaluation Procedure [34]. This means that, in order to improve the effectiveness of WASH interventions, data on facility functioning, and usage must be collected following implementation. Further research is required to evaluate not only adherence to the intervention but also the delivery method in an outbreak setting. This research will focus on cholera but there will be additional external benefits to be gained in the reduction of diarrhoeal diseases. Furthermore the WASH community as a whole is seeking leadership and guidance on how best to evaluate the health impact of their activities. Whilst implementation of the SPHERE standards has no doubt improved the quality of interventions this may have been at the expense of coverage. Different response agencies have vast practical experience of outbreaks, however, the lessons learned, of what works and why, remain unpublished, and evidence for best practice is not evaluated. To this end, we recommend that future research proposals are designed to be implemented as soon as an outbreak response is initiated. This means seeking pre-emptive funding commitments and ethics approval to avoid delays in the collection of baseline data which will be critical to sound evaluation. We call upon international agencies and institutions to integrate research protocols into their response strategy, and make the necessary funding and resources available. The results of this much needed operational research will be invaluable to informing international WASH policy, standards and practice with the ultimate aim being, to contribute to reducing the global burden of cholera.

## Supporting Information

S1 PRISMA Checklist PRISMA 2009 checklist. (DOCX)

S1 Table Search Strategy. (DOCX)

S2 Table Methodological Quality. (DOCX)
