# Supplementary material for: The Impact of Water, Sanitation and Hygiene Interventions to Control Cholera: A Systematic Review
Source: PLoS One. 2015 Aug 18;10(8):e0135676. doi: 10.1371/journal.pone.0135676 (PMC4540465; doi:10.1371/journal.pone.0135676)
Supplement: S2 Table — (DOCX) [file pone.0135676.s003.docx]

**Table S2 Methodological Quality**

| **No.** | **Study** | **Statistical Category (A, B, C)** | Intervention clearly described? | Target population defined? | Comparison group (e.g. baseline, control)? | Inclusion and exclusion criteria defined? | Sample size / method justified with statistical basis? | Statistical test (p-value or confidence interval)? | Adjustment for confounding? | Study limitations explained? | **STROBE SCORE** |
| --- | --- | --- | --- | --- | --- | --- | --- | --- | --- | --- | --- |
| **Water treatment at source** | |  |  |  |  |  |  |  |  |  |  |
| 1 | Cavallaro et al. (2011) | **C** | 1 | 0 | 0 | 0 | 0 | 0 | 0 | 1 | **2** |
| 2 | Garandeau et al. (2006) | **C** | 1 | 1 | 0 | 0 | 0 | 0 | 0 | 0 | **2** |
| 3 | Guevart et al. (2008) | **C** | 1 | 0 | 0 | 0 | 0 | 0 | 0 | 0 | **1** |
| **Water treatment at point of use** | |  |  |  |  |  |  |  |  |  |  |
| 4 | Colwell et al. (2003) | **A** | 1 | 1 | 1 | 0 | 1 | 1 | 0 | 0 | **5** |
| 5 | Conroy et al. (2001) | **A** | 1 | 1 | 1 | 0 | 0 | 1 | 0 | 0 | **4** |
| 6 | Deb et al. (1986) | **A** | 1 | 1 | 1 | 0 | 0 | 1 | 0 | 0 | **4** |
| 7 | Dunston et al. (2001) | **C** | 1 | 1 | 0 | 0 | 0 | 1 | 0 | 0 | **3** |
| 8 | Huq et al. (2010) | **A** | 1 | 1 | 0 | 1 | 1 | 1 | 0 | 0 | **5** |
| 9 | Lantagne & Clasen (2012) | **C** | 1 | 1 | 0 | 0 | 0 | 0 | 0 | 1 | **3** |
| 10 | Patrick, Berendes et al. (2013) | **C** | 1 | 1 | 0 | 0 | 1 | 1 | 0 | 1 | **5** |
| 11 | Quick, Venczel et al. (1996) | **C** | 1 | 1 | 1 | 0 | 0 | 1 | 0 | 1 | **5** |
| **Hygiene promotion** | |  |  |  |  |  |  |  |  |  |  |
| 12 | Beau de Rochars et al. (2011) | **C** | 1 | 1 | 0 | 0 | 0 | 1 | 0 | 1 | **4** |
| 13 | Einarsdottir et al. (2001) | **C** | 1 | 1 | 0 | 0 | 0 | 1 | 0 | 0 | **3** |
| 14 | Mahadik & Mbomena (1983) | **C** | 0 | 1 | 1 | 0 | 0 | 1 | 0 | 0 | **3** |
| 15 | Quick, Gerber et al. (1996) | **C** | 1 | 1 | 0 | 0 | 0 | 1 | 0 | 1 | **4** |
| **Water storage vessel disinfection** | |  |  |  |  |  |  |  |  |  |  |
| 16 | Steele et al. (2008) | **C** | 1 | 1 | 0 | 0 | 0 | 0 | 0 | 0 | **2** |
| **Household disinfection** | |  |  |  |  |  |  |  |  |  |  |
| 17 | Gartley, Valeh et al. (2013) | **C** | 1 | 1 | 0 | 0 | 0 | 1 | 0 | 1 | **4** |
| **Improved WASH infrastructure** | |  |  |  |  |  |  |  |  |  |  |
| 18 | Azurin & Alvero (1974) | **B** | 1 | 1 | 1 | 0 | 0 | 0 | 0 | 0 | **3** |

| **No.** | **Study** | Adequate control groups? | Dealt with one to one comparison, | Adequate control for confounding variables? | Health indicators recall? | Health indicator definition | Analysis by age | Record facility usage | Conclusion reflects results? | **Blum & Feachem SCORE** | **FINAL SCORE** | **QUALITY SCORE** |
| --- | --- | --- | --- | --- | --- | --- | --- | --- | --- | --- | --- | --- |
| **Water treatment at source** | |  |  |  |  |  |  |  |  |  |  |  |
| 1 | Cavallaro et al. (2011) | 0 | 0 | 0 | 0 | 0 | 0 | 0 | 1 | **1** | **1.5** | **low** |
| 2 | Garandeau et al. (2006) | 0 | 0 | 0 | 0 | 0 | 0 | 0 | 1 | **1** | **1.5** | **low** |
| 3 | Guevart et al. (2008) | 0 | 0 | 0 | 0 | 0 | 0 | 0 | 0 | **0** | **0.5** | **low** |
| **Water treatment at point of use** | |  |  |  |  |  |  |  |  |  |  |  |
| 4 | Colwell et al. (2003) | 1 | 1 | 0 | 0 | 0 | 0 | 1 | 1 | **4** | **4.5** | **med** |
| 5 | Conroy et al. (2001) | 1 | 0 | 0 | 1 | 1 | 1 | 0 | 1 | **5** | **4.5** | **med** |
| 6 | Deb et al. (1986) | 1 | 0 | 0 | 1 | 1 | 0 | 0 | 1 | **4** | **4** | **med** |
| 7 | Dunston et al. (2001) | 0 | 0 | 0 | 0 | 0 | 0 | 1 | 1 | **2** | **2.5** | **low** |
| 8 | Huq et al. (2010) | 0 | 0 | 0 | 1 | 1 | 0 | 1 | 1 | **4** | **4.5** | **med** |
| 9 | Lantagne & Clasen (2012) | 0 | 0 | 0 | 0 | 0 | 0 | 1 | 1 | **2** | **2.5** | **low** |
| 10 | Patrick, Berendes et al. (2013) | 0 | 1 | 0 | 0 | 0 | 0 | 1 | 1 | **3** | **4** | **med** |
| 11 | Quick, Venczel et al. (1996) | 1 | 1 | 0 | 0 | 0 | 0 | 1 | 1 | **4** | **4.5** | **med** |
| **Hygiene promotion** | |  |  |  |  |  |  |  |  |  |  |  |
| 12 | Beau de Rochars et al. (2011) | 0 | 1 | 0 | 0 | 0 | 0 | 1 | 1 | **3** | **3.5** | **med** |
| 13 | Einarsdottir et al. (2001) | 0 | 0 | 0 | 0 | 0 | 0 | 1 | 1 | **2** | **2.5** | **low** |
| 14 | Mahadik & Mbomena (1983) | 1 | 0 | 0 | 0 | 0 | 0 | 0 | 0 | **1** | **2** | **low** |
| 15 | Quick, Gerber et al. (1996) | 0 | 0 | 0 | 0 | 0 | 0 | 0 | 1 | **1** | **2.5** | **low** |
| **Water storage vessel disinfection** | |  |  |  |  |  |  |  |  |  |  |  |
| 16 | Steele et al. (2008) | 0 | 0 | 0 | 0 | 0 | 0 | 0 | 1 | **1** | **1.5** | **low** |
| **Household disinfection** | |  |  |  |  |  |  |  |  |  |  |  |
| 17 | Gartley, Valeh et al. (2013) | 0 | 0 | 0 | 0 | 0 | 0 | 1 | 1 | **2** | **3** | **low** |
| **Improved WASH infrastructure** | |  |  |  |  |  |  |  |  |  |  |  |
| 18 | Azurin & Alvero (1974) | 0 | 0 | 0 | 1 | 1 | 1 | 0 | 1 | **4** | **3.5** | **med** |
